# The Council of Emergency Medicine Residency Directors Academy for Scholarship Coaching Program: Addressing the Needs of Academic Emergency Medicine Educators

**DOI:** 10.5811/westjem.2018.9.39416

**Published:** 2018-11-13

**Authors:** Jaime Jordan, Michele L. Dorfsman, Mary Jo Wagner, Stephen J. Wolf

**Affiliations:** * University of California, Los Angeles, Ronald Reagan Medical Center, Department of Emergency Medicine, Los Angeles, California; † David Geffen School of Medicine at University of California Los Angeles, Department of Emergency Medicine, Los Angeles, California; ‡ University of Pittsburgh School of Medicine, Department of Emergency Medicine, Pittsburgh, Pennsylvania; § Central Michigan University Medical Education Partners, Department of Emergency Medicine, Saginaw, Michigan; ¶ Central Michigan University College of Medicine, Department of Emergency Medicine, Mt. Pleasant, Michigan; || Denver Health Medical Center, Department of Emergency Medicine, Denver, Colorado; # University of Colorado School of Medicine, Department of Emergency Medicine, Aurora, Colorado

## Abstract

**Introduction:**

Didactic lectures remain fundamental in academic medicine; however, many faculty physicians do not receive formal training in instructional delivery. In order to design a program to instill and enhance lecture skills in academic emergency medicine (EM) physicians we must first understand the gap between the current and ideal states.

**Methods:**

In 2012 the Council of Emergency Medicine Residency Directors (CORD) Academy for Scholarship designed a novel coaching program to improve teaching skills and foster career development for medical educators based on literature review and known teaching observation programs. In order to inform the refinement of the program, we performed a needs assessment of participants. Participants’ needs and prior teaching experiences were gathered from self-reflection forms completed prior to engaging in the coaching program. Two independent reviewers qualitatively analyzed data using a thematic approach.

**Results:**

We analyzed data from 12 self-reflection forms. Thematic saturation was reached after nine forms. Overall inter-rater agreement was 91.5%. We categorized emerging themes into three domains: participant strengths and weaknesses; prior feedback with attempts to improve; and areas of desired mentorship. Several overlapping themes and subthemes emerged including factors pertaining to the lecturer, the audience/learner, and the content/delivery.

**Conclusion:**

This study identified several areas of need from EM educators regarding lecture skills. These results may inform faculty development efforts in this area. The authors employed a three-phase, novel, national coaching program to meet these needs.

## INTRODUCTION

Despite multiple changes in medical education in recent years, didactic lectures remain a fundamental modality for instruction in academic medicine.^1^ However, many academic physicians lack formal training in instructional methods when assuming faculty positions. To meet this need, the creation and evolution of faculty development programs have helped faculty achieve specific skills.^2^ One example shown to be effective in improving teaching and lecturing skills in medicine is observation and feedback.^1^,^3^,^4^ Peer mentoring has also been shown to positively impact academic skills and professional development.^5^–^9^

Coaching has been described as a learner-centered method of evaluating performance, clarifying the meaning of outcomes and identifying strategies for success, with the ultimate goal of fostering insight and life-long learning skills.^10^ Coaching has been used in other fields to support professional development but only recently has emerged in medical education.^10^,^11^ Limited data suggest that coaching can improve clinical and teaching skills, enhance collaboration, decrease burnout, and positively impact professional development.^11^–^16^ Models for peer coaching in simulation debriefing and large-group teaching have been proposed.^17^,^18^

The Council of Emergency Medicine Residency Directors (CORD) sought to create a novel, national, faculty peer-coaching program to improve lecture skills and foster career development. The program was purposefully developed to take advantage of existing educational theories, including experiential learning, reflective learning, and deliberate practice.^19^–^21^ In order to inform program design and refinement, an understanding of the gap between the current and ideal states is essential. The objective of this study was to evaluate the needs and prior experiences of emergency medicine (EM) educators participating in the program.

## METHODS

We conducted a needs assessment of EM educators participating in the CORD Academy for Scholarship Coaching Program. The program was made available to all CORD members presenting at national meetings. Prior to participation, presenters completed a self-assessment form regarding their teaching experience and areas of desired mentorship (Appendix A). Responses were qualitatively analyzed using a thematic approach. Data were independently reviewed line by line by two investigators experienced in qualitative methods (JJ and SJW) to identify recurring concepts and assign codes, which were then further refined into themes using the constant comparative method.^22^ After independent review, the two investigators met to establish a final coding scheme that was applied to all data.

Analysis continued until thematic saturation was achieved, defined as no additional emerging themes.^23^ Discrepancies were resolved by in-depth discussion and negotiated consensus. This study was deemed “exempt” by the Central Michigan University Institutional Review Board.

## RESULTS

We analyzed data from 12 available self-reflection forms. Thematic saturation was reached after nine forms; however, we analyzed an additional three forms to ensure that no additional important themes were missed. Inter-rater agreement was 91.5%.

### Strengths and Weaknesses

Regarding strengths and weaknesses, as well as effective and ineffective teaching behaviors, three themes (factors pertaining to the lecturer, factors pertaining to the audience/learner, and factors pertaining to content and delivery) emerged. These themes were further broken down into 10 subthemes (Table 1). Generally, presenters felt that their lectures went well when they were well prepared, organized, spoke eloquently, effectively engaged their audience, highlighted the relevance of the information, aligned their content with education theory, incorporated active learning techniques, and optimally used audiovisual or supporting materials. Conversely, when they failed to do this, they felt their sessions were less effective. Presenters also noted challenges with larger groups and felt that their self-perception impacted their lectures.

### Prior Feedback and Attempts to Improve

Many presenters (7/12) had received positive feedback in the past. It is important to note that several commented on how they were motivated to improve and strive for excellence despite receiving this positive feedback. This sentiment is captured in the following statement:

This year, I won the New Speaker’s Forum at AAEM [American Academy of Emergency Medicine] and the Rising Star Award at ACEP [American College of Emergency Physicians]…However, I really do feel like there is room for improvement.

Themes for improvement were in line with what presenters had previously identified as weaknesses or ineffective teaching behaviors (Table 2). Regarding efforts that presenters had tried in order to improve their teaching, three major themes emerged: self-evaluation; informal education; and formal education.

### Mentorship Sought

Regarding desired mentorship, the majority (9/12) sought assistance in improving specific teaching skills. Multiple themes emerged that were congruent with identified weaknesses (Table 2). The most prominent subtheme that emerged was speaking style. Several participants remarked on their desire for excellence; for example, one presenter remarked:

I’ve given around a dozen or so national talks….I am looking for that ‘next level’ of improvement…I think I’m at the stage of being an ‘average national speaker’ and want to get to that ‘great speaker’ stage.

## DISCUSSION

In this study, EM educators identified multiple areas of need regarding lecture skills that were categorized into three major themes (factors pertaining to the lecturer, the audience/learner, and content and delivery), which can be used as a framework and organizational strategy for faculty development programs in this area. Within these major themes, multiple subthemes were identified. These subthemes can serve as specific areas for skill development. Several participants remarked on their desire for excellence, hoping to distinguish themselves nationally and improve their skills. This attitude lends itself well to the coaching model in which the goal is to help one achieve his/her personal best rather than a certain level of competency. Another interesting theme was self-perception. Several participants commented on the anxiety they experienced when lecturing and their lack of confidence. This highlights that psychological factors may have an impact on teaching ability. This is another opportunity where coaching can have a meaningful impact.

Based on the results of this study, the authors employed a three-phase coaching program to enhance teaching skills for medical educators involved in national speaking engagements:

- **Pre-observation phase**: The pre-observation phase serves as an opportunity for goal-setting and delineation of expectations for the presenter, coach, and the program. Presenters will complete a structured self-reflection form focusing on previous teaching experiences, feedback, and desired goals. In doing so, presenters engage in reflective practice and learning, setting the stage to build from previous experiences. Reflection has been shown in prior literature to have multiple benefits in medical education including increased learning, engagement, and comfort with difficult material.^24^ This reflection will guide a pre-observation meeting between the coach and presenter. The coach should note details in the specific content areas identified in this study (self-perception, preparation and knowledge, speaking style, engagement, relevance, large groups, alignment with educational theory, audiovisual and supporting material) provided by presenters and use this information to target observation efforts and frame post-observation feedback.
- **Observation phase**: The observation phase is directed at data collection. The trained coach critically observes the teaching session, attending to specific needs described in this study. Data can be collected as written observations, pictures, videos, or audio recordings. Significant emphasis should be placed upon documenting specific examples. Coaches need to be inconspicuous during their observation to assure that they are not affecting the delivery of the session.
- **Post-Observation phase**: This phase consists of a debriefing session where the coach leads a discussion of the opportunities for improvement, framed within the major themes highlighted in this study. The coach targets specific, desired skill areas outlined by the subthemes elucidated and by individual participant needs identified in the pre-observation session. This meeting should be reasonably proximate to the teaching session to minimize the impact of diminished recall. The coach should analyze his/her notes and develop formative themes before the debriefing. When leading this discussion, the coach should use multiple examples, allowing the feedback to be maximally meaningful and relevant.

The field of medicine embraces lifelong learning, and physician educators should strive to go beyond competency and achieve excellence. Coaching has the potential to be a valuable tool in faculty development for physicians at all stages of their careers. This novel, multi-institution, national faculty coaching program for lecture skills can address the perceived needs of EM physician educators.

## LIMITATIONS

This was an exploratory, qualitative needs assessment and the findings must be interpreted through that lens. The sample size was small and consisted of EM educators who desired coaching to improve their lecture skills; thus, the results may not be generalizable. Additionally, while thematic saturation was achieved, it is possible that important information was not captured in the analysis. Despite these limitations, the findings of this study may serve as an important foundation from which to build upon for how to improve the lecture skills of physician educators. Future, well-designed research studies evaluating objective outcomes of the program, such as lecture evaluations, number of invited speaking engagements, audience engagement, benefits to coach and presenter, and impact on professional development, are needed.

## CONCLUSION

This study identified several areas of need from EM educators regarding lecture skills including factors pertaining to the lecturer, the audience or learner, and the content and delivery. These results may inform faculty development efforts in this area. The authors support a three-phase, novel, national coaching program to meet these needs.

## Supplementary Material





## Figures and Tables

**Table 1:** Results of qualitative analysis regarding strengths and weaknesses of physician lecturers.

Theme	Subtheme	Number of comments	Exemplar quotes
Factors pertaining to the lecturer	Self-perception	12	“I generally consider myself to be an above average speaker.” “…Then, I begin to doubt myself and my talk which negatively impacts the talk.” “ …I get nervous in front of crowds in which I may not be the most expert person in the room…”
	Preparation and knowledge	11	“Seems to go well when I am well prepared, have in-depth knowledge of a subject, know my learners…” “I feel uncomfortable with spontaneous, ad lib, or dynamic settings.” “The last [lecture] I felt did not go well was clearly for a lack of preparation and rehearsal.”
	Speaking style	11	“Great command of language, cadence, and presence.” “…did all my usual talk tics: too frequent consultation of notes, [too many] ‘um’s’, long pauses, speaking too fast and too low…”
Factors pertaining to the audience/learner	Engagement	16	“…it opened with a personal story…so everyone’s attention was captured right away and I was able to form a connection with the audience.” “…it was harder to get the ‘connection.’ There were only a few in the front who were engaged.”
	Relevance	6	“…the information resonated with people.” “My strengths are in framing the importance of a problem…” “I felt the session went well because it is a topic that our medical students are rarely exposed to and thus highly motivated to learn about.” “… make points relevant to the residents, and make points that were relevant to our hospital.”
	Large groups	4	“I would like to develop techniques to help me do better with larger groups.”
Factors pertaining to content and delivery	Alignment with educational theory	6	“I try and focus on no more than 5 take-home points to keep the cognitive load manageable for the audience.” “The content was also very well matched to the knowledge level of the learners…”
	Audio visual/supporting material	7	“I used minimal slides, all of which had little to no text, so the attention was on me.” “My general teaching strengths are… the use of a whiteboard to visually present information.” “PowerPoint/presentation dedication and wizardry” “Some of [the low fidelity simulation] worked, but some of it didn’t….my slides were also too wordy…”
	Organization	10	“My lectures are well organized and present a central theme or story effectively.” “I can come across as disorganized at times.”
	Use of active learning techniques	7	“I try to use case-based methodology…” “Flipped classroom for initial information, synthesis of given material, active participation of audience in an activity.” “Teaching behaviors that worked well were the use of learner involvement through ‘think-pair-share,’ case-based activities and the building of comparison charts.”

**Table 2:** Results of qualitative analysis regarding prior feedback and desired mentorship.

Theme	Subtheme	Number of comments	Exemplar quotes
Prior feedback			
Factors pertaining to the lecturer	Preparation and knowledge	2	“More advanced prep.”
	Speaking Style	3	“My delivery may be too fast…or my speaking style may be too familiar.”
Factors pertaining to the audience/learner	Engagement	1	“I also get the sense I could do more as far as really engaging or entertaining the audience.”
Factors pertaining to content and delivery	Audio visual/supporting material	4	“I recently did give a talk and tested my audio-visual equipment ahead of time and still had problems.”
	Organization	2	“Constructive feedback: better organization”
Areas of desired mentorship			
Factors pertaining to the lecturer	Self-perception	2	“…to decrease my anxiety around giving talks…to be more comfortable speaking…Additionally, when I do receive feedback, I am working on how I interpret feedback and trying not to internalize and generalize negative feedback”
	Speaking style	8	“…I really haven’t gotten much feedback on technical aspects like how clearly I speak, the rate of which I speak, or how effectively I use the room space. This would also be helpful to get feedback on.”
Factors pertaining to the audience/learner	Engagement	3	“The big thing I want to work on is audience engagement…I could do more to draw the audience in….Are there ways in which I cannot just give a lecture but moderate an audience?”
	Large groups	2	“I would like to improve my ability to give a talk, particularly in a formal, large group setting.”
Factors pertaining to content and delivery	Audio visual/supporting material	2	“I would love feedback on my use of visual aids…”
	Organization	3	“I would like to improve specific teaching skills, particularly scaffolding/sequencing my learning materials…”
