# Supplementary material for: The Council of Emergency Medicine Residency Directors Academy for Scholarship Coaching Program: Addressing the Needs of Academic Emergency Medicine Educators
Source: West J Emerg Med. 2018 Nov 13;20(1):105–10. doi: 10.5811/westjem.2018.9.39416 (PMC6324694; doi:10.5811/westjem.2018.9.39416)
Supplement: Supplementary file 1 [file wjem-20-105-s001.pdf]

CORD Academy for Scholarship Coaching Program

Reflection Prior to Your Mentoring Session

This mentoring session is aimed at helping you to improve skills to achieve your goals. Consequently, it is important for you to take some time to think about your goals and current level of performance. We ask that you complete the following reflection to help clarify your thinking and better communicate with your mentor.

If you are unfamiliar with reflection, it is a process of critically analyzing, questioning, framing, and then reframing your experiences as a teacher to help you assess how you teach and to assist you in developing a plan to improve your teaching skills in ways that are important to you.

Please spend some time writing your responses to these questions. Use additional space as needed. Please return this form to your mentor via e-mail prior to your actual mentoring session.

1. **Consider a recent teaching session that you felt went well. Describe why you felt it went well, what teaching behaviors worked well and your general teaching strengths.**

2. **Consider a teaching session that did not go well. Describe why you felt it went poorly, what you thought, what teaching behaviors were ineffective and what you learned or would like to learn.**

3. What feedback have you received? What have you tried before to improve your teaching?

4. Consider the 3 mentoring domains listed below. Chose one and briefly elaborate the mentoring assistance you seek. Mentoring to help you . . .

a) understand your own needs and motivations for teaching;
b) improve specific teaching skills (e.g. lecturing, use of PowerPoints, small group or bedside teaching, the use of modeling and thinking out loud , scaffolding and sequencing your learning materials, use of Socratic questioning, or use of mentoring, reflection, and exploration as teaching tools);
c) develop other specific teaching competencies you may need (please describe).

Print Form
