# Supplementary material for: The Council of Emergency Medicine Residency Directors Academy for Scholarship Coaching Program: Addressing the Needs of Academic Emergency Medicine Educators
Source: West J Emerg Med. 2018 Nov 13;20(1):105–10. doi: 10.5811/westjem.2018.9.39416 (PMC6324694; doi:10.5811/westjem.2018.9.39416)
Supplement: Supplementary file 2 [file wjem-20-105-s002.docx]

Appendix B. Coaching Observation Instrument.

| CORD Academy Lecture Teaching Observation Feedback Form | | |
| --- | --- | --- |
| Instructor: | Observer: | |
| Course: | Date: | Time: |
| Any requested/specific areas of focus: | | |

|  | **Introduction** | **Notes** |
| --- | --- | --- |
| 1 | Introduced topic, stated goals, offered preview and established context |  |
|  |  |  |
| 2 | Gained attention and motivated learning |  |
|  |  |  |
| 3 | Established ground rules for participation |  |
|  |  |  |
|  | **Body of Lecture** | **Notes** |
| 1 | Organized around 3-5 main points or themes and presented them in clear fashion |  |
|  | . |  |
| 2 | Linked content to prior learning and provided relevance |  |
|  |  |  |
| 3 | Demonstrates command of the subject |  |
|  |  |  |
| 4 | Used PowerPoint, visuals, handouts or demonstrations to reinforce content |  |
|  |  |  |
| 5 | Varied presentation and provided opportunities for students to integrate material |  |
|  |  |  |
| 6 | Provided periodic summaries and linkages |  |
|  |  |  |
|  | **Conclusion** | **Notes** |
| 1 | Summarized key points w/o introducing new information |  |
|  |  |  |
| 2 | Provided conclusion or stimulated further thought |  |
|  |  |  |

|  | **Delivery** | **Notes** |
| --- | --- | --- |
| 1 | Exhibited enthusiasm and stimulated interest |  |
|  |  |  |
| 2 | Used appropriate voice, gestures, movement and eye contact |  |
|  |  |  |
| 3 | Repeated student questions so all could hear |  |
|  |  |  |
| 4 | Encourages audience interaction |  |
|  |  |  |
| 5 | Started and finished on time |  |
|  |  |  |
| 6 | Used lighting, microphone and other equipment effectively |  |
|  |  |  |

| **Notes:** | |
| --- | --- |
|  | |
|  | |
|  | |
|  | |
|  | |
|  | |
| **Strengths:** | **Recommendations:** |
|  |  |
|  |  |
|  |  |
|  |  |
|  |  |
|  |  |

| **Improvement / Follow-Up Plan:** |
| --- |
|  |
|  |
|  |
|  |
|  |
|  |
